# Integrating biological knowledge for mechanistic inference in the host-associated microbiome

**DOI:** 10.3389/fmicb.2024.1351678

**Published:** 2024-04-04

**Authors:** Brook E. Santangelo, Madison Apgar, Angela Sofia Burkhart Colorado, Casey G. Martin, John Sterrett, Elena Wall, Marcin P. Joachimiak, Lawrence E. Hunter, Catherine A. Lozupone

**Affiliations:** ^1^Department of Biomedical Informatics, University of Colorado School of Medicine, Aurora, CO, United States; ^2^Department of Integrative Physiology, University of Colorado, Boulder, CO, United States; ^3^Lawrence Berkeley National Laboratory, Environmental Genomics and Systems Biology Division, Biosystems Data Science Department, Berkeley, CA, United States

**Keywords:** microbiome, databases, ontologies, inference, microbiology, computational biology

## Abstract

Advances in high-throughput technologies have enhanced our ability to describe microbial communities as they relate to human health and disease. Alongside the growth in sequencing data has come an influx of resources that synthesize knowledge surrounding microbial traits, functions, and metabolic potential with knowledge of how they may impact host pathways to influence disease phenotypes. These knowledge bases can enable the development of mechanistic explanations that may underlie correlations detected between microbial communities and disease. In this review, we survey existing resources and methodologies for the computational integration of broad classes of microbial and host knowledge. We evaluate these knowledge bases in their access methods, content, and source characteristics. We discuss challenges of the creation and utilization of knowledge bases including inconsistency of nomenclature assignment of taxa and metabolites across sources, whether the biological entities represented are rooted in ontologies or taxonomies, and how the structure and accessibility limit the diversity of applications and user types. We make this information available in a code and data repository at: https://github.com/lozuponelab/knowledge-source-mappings. Addressing these challenges will allow for the development of more effective tools for drawing from abundant knowledge to find new insights into microbial mechanisms in disease by fostering a systematic and unbiased exploration of existing information.

## 1 Introduction

The structure and function of the human microbiome can be both a driver and consequence of various disease states (Falony et al., 2019; King et al., 2019). Microbiome signatures are associated with a range of conditions including auto-immune and gastrointestinal disease, cancer, and neurological disease (Berg et al., 2020). Understanding interactions between the gut microbiome and the host at a mechanistic level requires a sophisticated synthesis of individual microbial functions, such as metabolic output, and how these functions interact with host processes that influence human physiology.

Mechanistic prediction of microbe-host interactions often involves in-depth analyses of multi-omic datasets. For example, in one study that related immune markers, microbiome composition, metabolomic data, diet, and demographic measures to markers of metabolic health in people living with human immunodeficiency virus (HIV), they found that butyrate production and mucolytic activity of particular gut microbes play a potential role in intestinal barrier dysfunction, suggesting more targeted followup studies (Armstrong et al., 2021). In another study that used metagenomic, metatranscriptomic, metaproteomic, and metabolic data to explore the functional attributes of the microbiome that influence Parkinson’s disease (PD) pathogenesis, a preliminary result found the metabolite 2-hydroxypyridine (2-HP) and the microbe *Methanobrevibacter smithii* to be enriched in PD, prompting several experiments which verified their effects on alpha synuclein aggregation (Wilmes et al., 2022). Large scale metagenomics studies can hone in on these interactions at a species or strain level, and bioinformatics analyses can further hypothesize how the microbial community contributes to health or disease (Armour et al., 2019; Wallen et al., 2022). Such studies provide rich information in the scientific literature on associations between microbes, host pathways and diseases, which brings us closer toward a mechanistic understanding of the microbiome. Despite advances in bioinformatics techniques to evaluate multi-omic datasets and laboratory methods to further explore promising results, there is no efficient and reliable solution for using existing knowledge to identify the most promising potential mechanisms involving the microbiome in human disease for further experimental validation.

**Figure 1:**
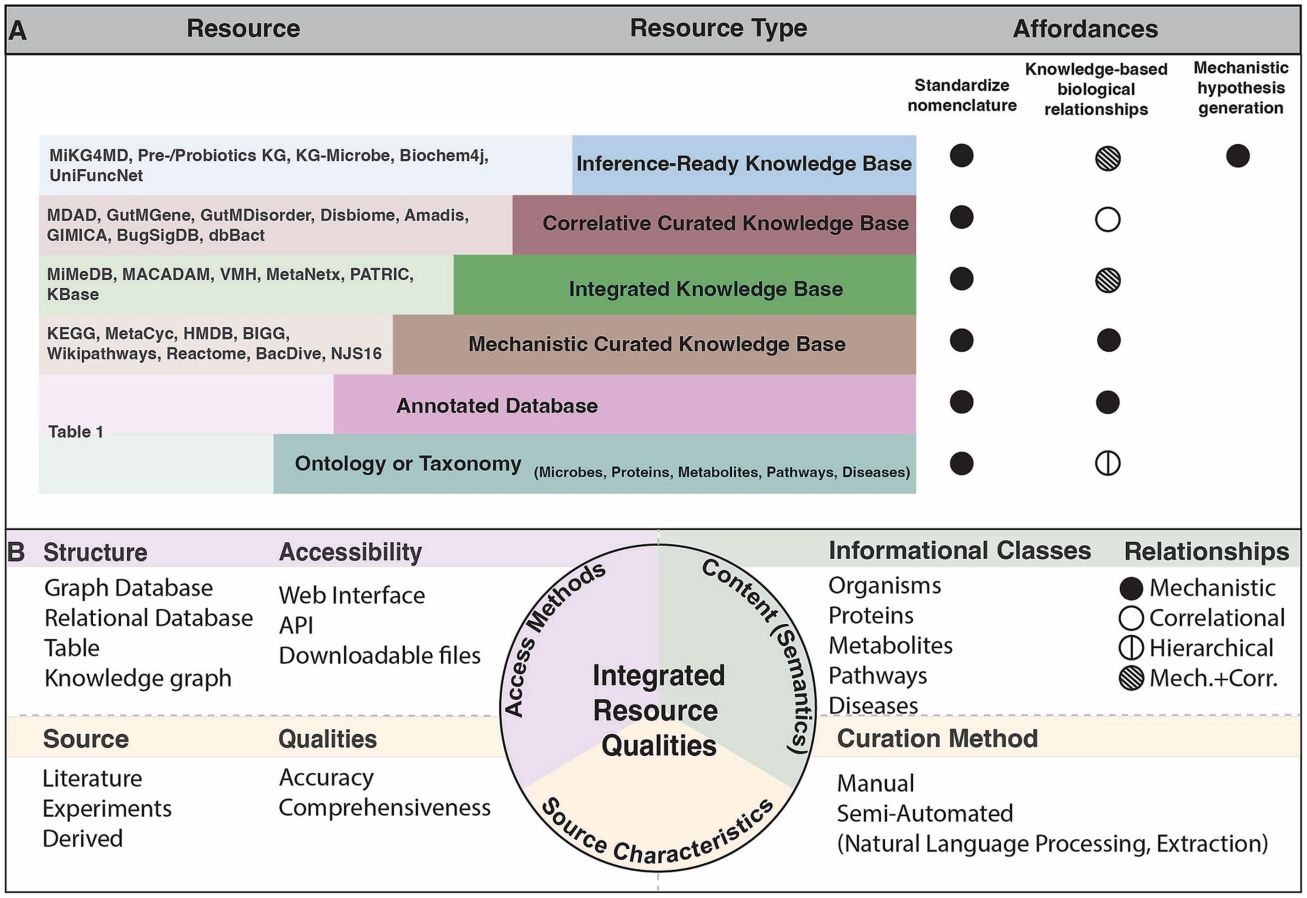
Characterization of known resources relevant to microbiome research. **(A)** Schematic of the types of resources that exist and the purpose that they serve in microbiome research. Note that resource characterization is based on the prominent qualities, though many resources span these types. Affordances represent the primary purpose of the given resource type. The standardized nomenclature affordance indicates that the resource introduces new identifiers to uniquify concepts. The knowledge-based biological relationships affordance implies that the resource describes interactions among the concepts by the indicated relationship type in **(B)**. The mechanistic hypothesis inference affordance indicates that the resource is uniquely suited to provide a mechanistic explanation when given specific queries. **(B)** The evaluations performed over existing resources mentioned in the Resources column of **(A)** within this review.

Public resources that organize microbial knowledge serve an important purpose in mechanistic inference. These can be summarized into six categories, with relevant concepts defined in Box 1: (1) *Ontologies and taxonomies* which provide a standardized nomenclature and hierarchical ranking of biological entities such as microorganisms, proteins or metabolites, (2) *Annotated databases* that have some information about the given concept that is linked to an experimental result, (3) *Mechanistic curated knowledge bases* that contain knowledge drawn from multiple data sources and render known explanations about biological interactions (4) *Integrated knowledge bases* which aggregate relationships and identifiers represented across many different sources (5) *Correlative curated knowledge bases* which include associations found between two unique concept types, e.g., a microbe and a disease, and (6) *Inference-ready knowledge bases* that enable mechanistic inference (Figure 1A). Synthesizing microbial and host information is critical to achieve a systems level perspective of the microbiome. Such integrated resources elucidate relationships between microbes and other biological concepts, allowing researchers to access the increasing amount of information to draw new conclusions.

BOX 1 **Key terms**

- Data repository: an archive of any data formats to enable public sharing
- Primary knowledge source: a source of knowledge that is used as a nomenclature standard for a knowledge base, for example an annotated database or ontology
- Ontology or Taxonomy: a system that is used as a semantic standard with a hierarchical classification scheme approved by groups of experts (Carpendale et al., 2014)
- Annotated database: database that stores experimentally derived content, such as sequences or structures from an instrument, with data labels potentially from manual curation
- Knowledge Base: structured repository describing the relationships between categories and the standardizing mappings of such categories*
- Mechanistic knowledge: an assertion of causal relationships between two categories
- Correlative knowledge: an assertion of statistical associations between two categories
- Mechanistic curated knowledge base: knowledge base derived from manual curation over multiple knowledge sources
- Correlative curated knowledge base: repository of correlative knowledge derived from manual curation over multiple knowledge sources
- Integrated knowledge base: knowledge base that incorporates content from multiple knowledge sources, most often cross-linking identifiers over such resources
- Inference-ready knowledge base: knowledge base that represents relationships in a logically consistent and semantically well-defined manner

*A category here signifies a class of knowledge based on empirical evidence, often referred to as a concept or entity. The three terms are used interchangeably throughout this review.

We evaluate the accessibility of the many resources that fall within these categories, which alludes to the structure that the content is made available in, and the interfaces that users are able to access the resource. The various ways that knowledge bases are made available, whether through downloadable files, programmatically, or via a user interface, influences how useful they are among scientists. We identify the content of each resource including the types of information classes that are represented and the types of relationships between concepts. Lastly, we critique the source characteristics of each resource by assessing the source of knowledge, the curation method, and the qualities such as accuracy that result from those curation methods. We assess comprehensiveness and accuracy by examining how each resource was constructed and how automated processes can lack the specific or validated evidence provided by manual curation. The most effective integrated resources are those which link all categories of knowledge, and align the concepts represented to identifiers of ontologies or primary knowledge bases (Figure 1B). These resources thereby allow for sophisticated computational analyses and inference to understand microbial mechanisms. In this review, we assess how integrated resources and tools can be used to address mechanistic questions in microbiome research.

## 2 Efforts to standardize microbiome studies

Understanding the host-associated microbiome is particularly challenging given the need to incorporate both microbial and host processes into analyses. Due to the interdisciplinary nature of microbiome research, there have been many efforts to develop broad standardization of experimental design, metadata, and reported results of observational and genetic studies in the field. The Genomic Standards Consortium (GSC) introduced two important standards: minimum information about any (x) sequence (MIxS) and minimum information about marker gene sequence (MIMARKS), and a checklist for microbiome study reporting and manuscript preparation (Yilmaz et al., 2011; Mirzayi and Renson, 2021). Platforms such as Qiita, which allows users to perform microbiome analyses for one or more studies, require the metadata to be entered according to MIxS standards (Gonzalez et al., 2018). These standards ensure consistency in reporting metadata of new, published experimental results and support integration of data across studies seamlessly.

In addition to standardizing metadata, it is important to unify the representation of concepts for multi-omic studies. Integrated resources harmonize biological content by mapping entities to standardized ontologies or other primary databases. The nomenclature of microbes, proteins, and metabolites that are involved in a microbiome study may vary, and aligning these terms is important for aggregation. It is most useful if the concepts represented are mapped to universally accepted identifiers such as ontologies or taxonomies (Box 1). Many domain specific ontologies exist in the Open Biological and Biomedical Ontology Foundry (OBO) that are widely relevant to biomedicine, such as the Gene Ontology (GO) that provides a directed acyclic graph (DAG) structure to the biological processes, cellular components, and molecular functions that result from gene products (The Gene Ontology Consortium, 2019; Jackson et al., 2021). Structured databases that consolidate external annotations, align nomenclature, and provide frequent updates can also be the main source of identifiers. PubChem is one such resource of chemical information including molecular formula, structure, and physical properties, while DrugBank expands on this information to include drug target sequences and pharmacological properties (Wishart et al., 2018; Kim et al., 2023). This process of standardization thus enables the contextualization of specific experimental results to a broader class of concepts (chemicals, proteins, organisms, etc.). The primary databases relevant for microbiome research are described in Table 1. These efforts for standardization of both metadata and microbial concepts have supported the development of integrated resources that combine functional and metabolic concepts of microbes and the host.

**Table 1:** Primary knowledge sources for the standardization of all entity types.

(A) Microbial classification resource	Nomenclature	Trait based	Sequence based	*De novo* tree based	Update frequency
*Ontologies and taxonomies*					
Bergey’s Manual of Systematic Bacteriology (Goodfellow et al., 2009)	X	X			2–4 y
NCBI Taxonomy (Federhen, 2012)	X	X			6 months
*Deutsche Sammlung fur Mikroorganismen und Zellkultren (DSMZ)*	X		X		1–4 months
Genome Taxonomy Database (GTDB) (Parks et al., 2022)	X		X		6 months
Greengenes, Greengenes2 (DeSantis et al., 2006; McDonald et al., 2023)	X		X	X	Irregular
SILVA (Quast et al., 2012)	X		X	X	1–2 years
Unified Medical Language System (UMLS) (Bodenreider, 2004)	X				2.5 months
Systematized Nomenclature of Medicine-Clinical Terminology (SNOMED CT) (Vuokko et al., 2023)	X				1 year
Medical Subject Headings (MeSH)	X				1 year

(B) Functional characterization of genes resource	Nomenclature	Sequence	Function	Homologous Groupings	Microbe Oriented	Host Oriented	Update frequency
*Ontologies and taxonomies*							
Protein Ontology (PRO) (Chen et al., 2020)	X				X	X	2–6 months
Gene Ontology (GO) (The Gene Ontology Consortium, 2019) – subsumes EC			X		X	X	1 month
*Annotated Databases*							
Protein Data Bank (PDB) (wwPDB consortium et al., 2019)	X				X	X	1 week
SWISS-PROT/Trembl (Boeckmann, 2003)	X	X			X	X	1 month
Cluster of Orthologous Groups (COGs) (Galperin et al., 2021)	X	X		X	X	X	Irregular
InterPro/Pfam (Paysan-Lafosse et al., 2023)	X	X	X	X	X	X	1–3 months
Carbohydrate Active Enzymes (CAZy) (Cantarel et al., 2009)	X	X	X	X	X	X	1 month
GenBank (Sayers et al., 2021)	X	X			X	X	2 months
RefSeq (O’Leary et al., 2016)	X	X			X	X	1 year
Entrez (Maglott et al., 2007)	X	X					Daily
Ensembl (Howe et al., 2021)	X	X					0.5 months
Protein Information Resource/Protein Sequence Database (PIR/PSD) (Barker et al., 2000)	X	X			X	X	3 months
Protein Extraction, Description and ANalysis Tool (PEDANT) (Frishman, 2003)	X	X			X	X	Irregular
microRNA sequence database (MiRBase) (Griffiths-Jones, 2006) (host oriented only)	X	X				X	1 year
Universal Protein Resource Knowledge Base (UniProtKB) (The UniProt Consortium et al., 2023) *note this contains SWISS-PROT/Trembl	X	X	X		X	X	1 month
AnnoTree (Mendler et al., 2019) *note this contains InterPro annotations (microbe oriented only)	X	X	X		X		Dep. on GTDB
Bacterial and Viral Bioinformatics Resource Center (BV-VRC) (Olson et al., 2023) (microbe-oriented only)	X	X			X		Irregular
Functional Annotation of Prokaryotic Taxa (FAPROTAX) (Liang et al., 2020)	X	X	X	X	X		Irregular
Enzyme Commission (EC) (Biochemistry IU of Committee MBN and Webb, 1992)	X		X		X	X	2.5 months
BRaunschweig ENzyme DAtabase (BRENDA) (Chang et al., 2021)	X	X	X		X	X	6 months
EggNOG (Huerta-Cepas et al., 2019)	X	X	X	X	X	X	2–3 y
SEED (Overbeek et al., 2014)	X	X	X	X	X	X	Dep. on resources

(C) Metabolite and reaction classification resource	Nomenclature	Gene interaction	Chemical reaction	Microbe oriented	Host oriented	Update frequency
*Ontologies and taxonomies*						
Chemical Entities of Biological Interest (ChEBI) (Hastings et al., 2016)	X			X	X	1 month
Chemical Function Ontology (Wishart et al., 2023)	X	X		X	X	New
*Annotated Databases*						
ChEMBL (Zdrazil et al., 2023)	X	X	X	X	X	6 months
PubChem (Kim et al., 2023)	X			X	X	1 year
SABIO-Reaction Kinetics Database (Wittig et al., 2018)	X		X	X	X	1 year
DrugBank (Wishart et al., 2018)	X	X		X	X	1 year
Rhea (Bansal et al., 2022)	X		X	X	X	2 months

(D) Pathway classification resource	Microbe oriented	Host oriented	Update frequency
*Ontologies and taxonomies*			
Pathway ontology (Petri et al., 2014)	X	X	1 week
Small Molecule Pathway Database (SMPDB) (Jewison et al., 2014)		X	2–4 years
PathBank (Wishart et al., 2020)	X	X	2–4 years

(E) Disease classification resource	Nomenclature	Disease classification	Update frequency
Ontologies and taxonomies			
Disease Ontology (Schriml et al., 2022)	X	X	1 year
Monarch Disease Ontology (Vasilevsky et al., 2022)	X	X	1 month
Unified Medical Language System (UMLS) (Bodenreider, 2004)	X		6 months
Systematized Nomenclature of Medicine-Clinical Terminology (SNOMED CT) (Vuokko et al., 2023)	X		1 year
Medical Subject Headings (MeSH)	X		1 year
Chemical Function Ontology (Wishart et al., 2023)	X		New
International Classification of Diseases (ICD) (Harrison et al., 2021)	X		1–4 years

The Nomenclature column specifies if the primary knowledge source provides identifiers for concept names. (A) Primary knowledge bases for microbial classification. Trait based resources use inherent traits, structural or otherwise, to differentiate taxa. Sequence-based resources rely on the genomic content, and *de novo* tree-based resources use some sequence-based and some machine learning techniques to differentiate taxa. All microbial classification resources are microbe-oriented. (B) Primary resources for functional characterization of microbial genes. Primary resources may link a given protein with its genomic sequence (Sequence), describe the function of a protein (Function), or describe the evolutionary relationships among proteins (Homologous Groupings). (C) Primary knowledge bases used for metabolic modeling. Primary resources may link a given chemical with a target genomic sequence (Gene Interaction) or describe the reactions that a chemical is involved in (Chemical Reaction). (D) Primary knowledge sources used for pathways. (E) Primary knowledge sources used for diseases. Primary resources that include a hierarchical classification of diseases are noted (Disease classification). All disease resources are host-oriented.

## 3 Microbiome-relevant knowledge bases and their applications

**Figure 2:**
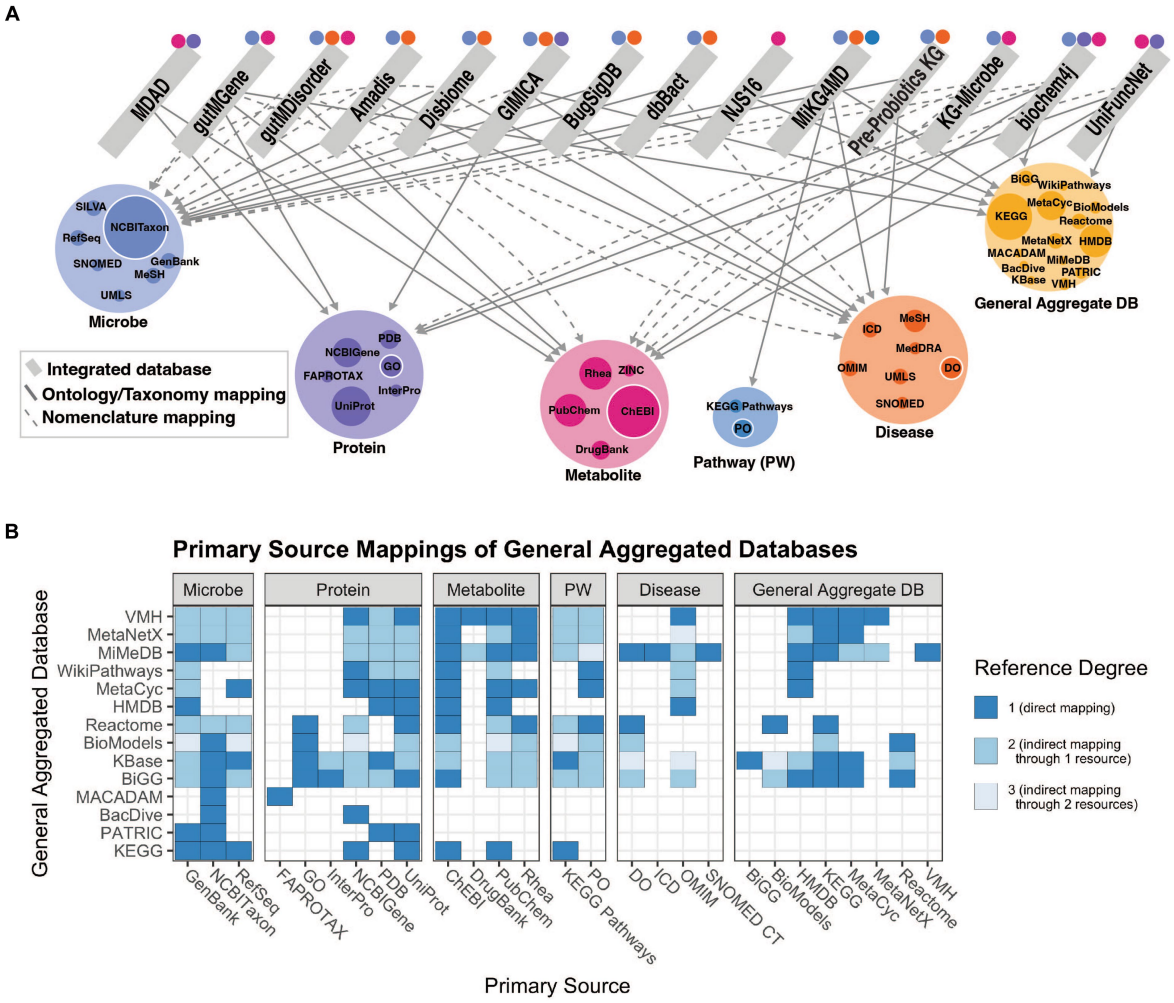
**(A)** Network of relationships included in integrated resources. Edges between an integrated resource and some type of primary knowledge source (microbe, protein, metabolite, pathway, or disease) represent either a categorization of the concept via an ontology or taxonomy (solid lines), or a nomenclature mapping to some identifier (dashed lines). Node size of the primary knowledge source (colored) represents the in-degree from integrated databases, where the largest nodes are those most often used for standardization. Colored points above integrated resources specify to which concept type the integrated resource maps, including indirect mappings through a general aggregate database. **(B)** Relationships among all general aggregate databases and primary databases, separated by category. Reference degree shows the degree to which a primary database may be referenced, indicating those most often used for standardization. E.g., If database i references another database *j*, a general aggregate database that in turn references database *k*, then *i* and *k* have a reference degree of 2. That primary database is only referenced if shown in **(A)**. This figure was generated using code and data available in github repository: https://github.com/lozuponelab/knowledge-source-mappings.

Our understanding of the microbiome is improved through knowledge of the relationships between individual microbial taxa, the functional characterization of their genes and how genomic content contributes to their metabolic outputs, and other information on microbial traits and functions determined through experimentation. Relating microbial taxonomic and functional information to host pathways, physiology or disease can provide mechanistic detail that informs our understanding of microbe-host interactions. This knowledge is made available through methods to collect and curate knowledge of microbial functions from the literature using natural language processing or manual annotation and representing the information in the form of integrated resources. We assess the relationships among integrated knowledge bases and their mappings to primary databases in Figure 2. The varying categories of integrated resources are highly applicable to three primary use cases: effectively accessing systems level microbiome information, contextualizing new findings with existing findings, and inferring new relationships to better understand how microbes influence disease. More detail of these resource qualities is described in Supplementary Table 1.

### 3.1 Knowledge bases that streamline access

An increasing number of knowledge bases have been developed that synthesize microbial and host content for systems biology research, including mechanistic curated knowledge bases and integrated knowledge bases. The relationships represented capture biological processes in a causal way, and are rooted in human curation of specific, validating experiments. In most cases, these resources introduce new unique identifiers for informational classes, which, in combination with other primary databases discussed previously, supports standardization and integration of correlative and inference-ready knowledge bases. In order to connect microbial sequences from experimental studies to these resources, sequence search tools such as Basic Local Alignment Search Tool (BLAST) or functional annotation tools such as InterPro and EggNog Mapper are used, alongside additional web applications such as MolEvolvR facilitating protein characterization across phylogenetic contexts (McGinnis and Madden, 2004; Jones et al., 2014; Cantalapiedra et al., 2021; Krol et al., 2022).

#### 3.1.1 Resources that include microbial and host genes, reactions, pathways, and metabolites

The Kyoto Encyclopedia of Genes and Genomes (KEGG) and MetaCyc are examples of mechanistic curated knowledge bases that represent relationships among microbial and host genes, reactions/pathways, and metabolites (Kanehisa et al., 2017; Caspi et al., 2020). KEGG and MetaCyc provide direct taxonomic mappings to NCBI Taxonomy, RefSeq, or GenBank, as well as direct mappings to multiple primary sources of metabolites and proteins (Figure 2). Both knowledge bases integrate knowledge of metabolic pathways for many organisms (Mendoza et al., 2019; Caspi et al., 2020). These knowledge bases organize pathway and molecular content in unique ways, often making comparison difficult, however a primary difference is in the supported tools surrounding the resource. KEGG introduces several tools including the BlastKOALA (short for KEGG Orthology And Links Annotation), a protein annotation web service, KEGG Mapper, a genome annotation service, and Pathogen Checker, a service supporting search of antimicrobial resistance genes (Kanehisa et al., 2017). KEGG also introduced drug and disease links to the pathways represented in 2005 and 2008, respectively. MetaCyc introduces Pathway Tools consisting of key components such as PathoLogic, a method to predict metabolic pathways of a given organism, and MetaFlux to generate genome-scale metabolic networks (GSMNs or GEMs) using flux based analysis (Caspi et al., 2020). Each of these platforms support extensive web-based interfaces for exploring the content represented, and both have moved to a subscription model. The large diversity of life represented among MetaCyc and KEGG render them broadly relevant to understanding microbiome-related results.

#### 3.1.2 Resources that host GSMNs

A method that systematically evaluates microbial phenotypes relevant for microbe:host interactions is GSMNs. GSMNs are *in silico* models and can be used to infer metabolic phenotypes. GSMNs use the annotated genes of an organism, which describe the associated biochemical reactions that the enzymatic products of such genes are capable of affecting. These gene annotations are found using annotation tools such as GapMind or aggregated from publicly available curated databases (Price et al., 2020). GSMNs serve two purposes: they synthesize knowledge of that organism’s metabolism, and they are a mathematical model which can be used to simulate metabolic phenotypes in environments of interest (Moretti et al., 2021). Moreover, GSMNs from multiple organisms can be aggregated in order to simulate entire microbial communities with tools such as MICOM (Swainston et al., 2017). Recently, MICOM was used to predict the risk of *Clostridium difficile* infection, the leading cause of antibiotic associated diarrhea, based on the metabolic strategies of *C. difficile* in different host microbiome and diet contexts (Carr et al., 2023). GSMNs can therefore serve as a blueprint for the suite of metabolic transformations possible and facilitate the understanding of the metabolic potential of a given microbial community (Mendoza et al., 2019; Esvap and Ulgen, 2021; Passi et al., 2021).

GSMNs are highly dependent on knowledge sources used in their construction. Because many different methods to generate GSMNs exist and it is often a manual process, there are often inconsistencies in the resulting models (Heinken et al., 2023). These differences are influenced by the reconstruction approach and attributed to the chosen database or annotation tools from which the information is gathered (Magnúsdóttir et al., 2017; Machado et al., 2018; Hsieh et al., 2023). The consistent mapping of the biological concepts represented in public databases and knowledge bases is a critical aspect of their broad utility. This standardization challenge expands beyond GSMNs to all resources that combine unique forms of knowledge based on prior studies, and remains a major limitation in the causal mechanism generating task.

There are several key resources hosting GSMNs, including the Biochemical, Genetic and Genomic knowledge base (BiGG), MetaNetX, BioModels, the Department of Energy Systems Biology Knowledgebase (KBase), and the Virtual Metabolic Human (VMH) (Arkin et al., 2018; Malik-Sheriff et al., 2019; Noronha et al., 2019; Norsigian et al., 2019; Moretti et al., 2021). BiGG enables an efficient search over multiple GSMNs by integrating published models of different organisms with standardized nomenclature of all components, with models of 108 organisms included as of 2019(Norsigian et al., 2019). BiGG and BioModels make high-quality GSMNs available to the academic community. Over the years, these have been updated to introduce features such as including genome annotations, standardizing reactions and metabolites to primary sources, and a greater taxonomic diversity of models (Malik-Sheriff et al., 2019; Norsigian et al., 2019). The VMH connects human metabolism, genetics, and disease with microbial metabolism and diet. The VMH cross-references over 57 resources to combine GSMNs of humans and microbes drawn from existing metabolic maps, experimental data from literature, and other integrated resources including BiGG (Noronha et al., 2019). VMH is a useful resource for studies seeking available knowledge of metabolite profiles. For example, the VMH was used in an evaluation of the influence of the Mediterranean diet on aging and the gut microbiome (Ghosh et al., 2020). MetaNetX is an integrated knowledge base that provides a mapping between major GSMN databases for more standardized representation of metabolic processes (Moretti et al., 2021). The goal of this resource is to reconcile the biochemical and metabolic content represented in key public databases. MetaNetX both provides cross-links and merges equivalent biochemical reactions and metabolites into a single identifier, such that entities are merged based on reaction context or chemical formula (Moretti et al., 2021). MetaNetX provides a straightforward way to access the relationships among metabolites through many GSMN sources. KBase is a resource funded by the US Department of Energy that integrates external repositories with data generated on the system, e.g., for public access to genomes and their corresponding metabolic models, including KEGG, BiGG, and MetaCyc, thus including metabolic models for 773 gut microbes as of 2018 and potentially more GSMNs that are currently private (Arkin et al., 2018). KBase also supports a suite of tools that allow for the construction of these metabolic models and workflows supporting the assembly of genomes all the way through to metabolic reconstruction, as well as many other computational tools for omic analyses. These user generated tools can generate data linkages to Functional Annotation of Prokaryotic Taxa (FAPROTAX) and InterPro, and all database entries and mappings are inherited from ModelSEED (Arkin et al., 2018; Seaver et al., 2021).

#### 3.1.3 Resources that host microbe and host metabolic content

There are several curated and integrated knowledge bases focused on centralizing known metabolic traits and output in host and microbial environments. The Human Microbial Metabolome Database (MiMeDB) connects microbial and human metabolism among many resources, including the VMH, as well as genome or proteome information with a focus on how the human microbiome influences health (Wishart et al., 2023). While MiMeDB represents fewer diseases than KEGG, they are constrained to those that are understood to be affected by microbial metabolism. MiMeDB furthermore supports specific constraints on the search of all entity types within the web-interface (e.g., co-metabolite, microbial, or human metabolite type). The MetAboliC pAthways DAtabase for Microbial taxonomic groups (MACADAM) is focused on functional annotations and integrates pathway genome databases (PGDBs) from MetaCyc, MicroCyc, FAPROTAX, and International Journal of Systematic and Evolutionary Microbiology (IJSEM) with genomes from RefSeq (Le Boulch et al., 2019). The Human Metabolome Database (HMDB) has accelerated the standardization of metabolic output and originally provided a uniquely centralized resource of broadly relevant human metabolomic data (Wishart et al., 2022). In 2021 microbial or gut-derived metabolites were added to the HMDB, supporting disease-focused investigation of microbial pathways. With the comprehensive array of metabolites documented, the known metabolite-disease associations in HMDB were used in a deep learning method intended to predict novel disease associated metabolites (Sun et al., 2022). The HMDB ecosystem also introduces tools such as DeepMet, a deep generative model for identifying new metabolites and potential hypotheses surrounding them (Wishart et al., 2022). Other deep learning based methods for understanding microbe-metabolite relationships include MMVec, BiomNED, and MiMeNet (Morton et al., 2019; Le et al., 2020; Reiman et al., 2021).

#### 3.1.4 Resources that include microbial trait and genomic content

Curated databases that incorporate microbial trait information or genomic content can illuminate functional qualities of microbes. These include the Bacterial Diversity Metadatabase (BacDive) and the Pathosystems Resource Integration Center (PATRIC) (Gillespie et al., 2011; Söhngen et al., 2014). PATRIC integrates genomic, transcriptomic, protein–protein interaction, protein structure, and other diverse data types for 22 genera of prokaryotic bacteria, mainly pathogens (Gillespie et al., 2011). This integrated knowledge base, which also includes some correlative results of host-pathogen-disease associations, compiles this information from publicly available datasets for users to easily view and analyze such results. BacDive is the largest standardized resource of prokaryotic information, consisting of strain level details of phenotypes, morphology, growth patterns, metabolism, and sequences for over 70,000 strains (Söhngen et al., 2014).

#### 3.1.5 Resources that include microbial or host pathway content

Several graph relational databases exist that support more complex queries based on their structure. These resources incorporate semantically defined relationships between concepts at a much greater depth than those represented in KEGG or MetaCyc. The Reactome Knowledgebase (Reactome) is a graph database that synthesizes human molecular processes in a standardized way such that all concepts are rooted in ontologies or primary databases (Fabregat et al., 2018). With over 10,000 human genes and their function incorporated, Reactome provides a high-level metabolic map for the interaction between the genome, the proteome, and the metabolome in humans. Reactome is not as broadly relevant to the specific microbe-human interactions that exist elsewhere as only pathogenic bacteria and infectious diseases are included. WikiPathways is another graph database of biological pathway models for all species, though mostly focused on human biology (Martens et al., 2021). Reactome and WikiPathways are community driven, which results in content that reflects the current consensus and supports more frequent updates. Reactome and WikiPathways provide interactive network visualizations of curated processes and pathways for the user to browse the concepts represented.

### 3.2 Contextualizing experimental findings

Whereas the previous section described curated and integrated knowledge bases that allow scientists to effectively access systems-level microbiome information, a second category of knowledge bases represent literature findings. Correlative knowledge bases allow researchers to contextualize new findings with existing findings in the literature, such as previous studies that have detected a relationship between a microbe and a disease, pathway, or other entity through laboratory or population level studies. These resources organize previously found associations between microbes and other entities, making knowledge computationally accessible.

In response to the growing number of drug resistant bacteria, the Microbe-Drug Association Database (MDAD) was built through manual curation of literature describing microbe-drug relationships based on PubMed keywords (Sun et al., 2018). The studies represented are either microbe-drug relationships identified through lab experiments or those found effective in clinical trials. GutMGene is another database created after manually searching PubMed articles for evidence of associations between microbes and metabolites produced or consumed, and microbial influence on human gene expression (Cheng et al., 2022). GutMDisorder similarly synthesizes associations between microbes and human diseases or phenotypes found in the literature (Cheng et al., 2020). Disbiome contains microbe-disease associations found from population level studies that identified significant differences in abundance between a control and disease state (Janssens et al., 2018). Amadis similarly provides evidence of associations between diseases and microbes, with a similar number of disease entries as Disbiome (Amadis includes relationships between 221 human diseases and 774 microbes, while Disbiome includes 190 human diseases and 800 microbes) (Janssens et al., 2018; Li et al., 2021). The Host Genetic and Immune Factors Shaping Human Microbiota (GIMICA) is another database representative of multiple human body sites and the immune, environmental, and genetic factors that they interact with (Tang et al., 2021). Several other link-based aggregate databases introduced more stringent manual curation techniques to adequately represent the variable aspects of studies, such as experimental setting or sequencing technique. BugSigDB is a community-supported effort of over 2,500 curated microbial signatures cited in over 600 scientific articles. With over 1,400 unique taxa represented, BugSigDB is rich in metadata, experimental conditions, and design of each experiment and is well standardized to a range of ontologies. Another knowledge base, dbBact, includes over 900 experiments and supports similar use cases aligning results across many studies (Amir et al., 2023). These literature-based databases support easy access to information in a context dependent manner. The provenance of such associations is also easily made available within these resources by PubMed Identifier (PMID). NJS16 is an integrative network that incorporates manually curated knowledge from literature of gut microbes and how they interact via metabolite transport (Sung et al., 2017). NJS16 uses a metagenomic analysis of a cohort of Type 2 Diabetes individuals to showcase a framework that can predict microbe-metabolite interactions that influence host physiology in other contexts. Such manually curated resources play a critical role in allowing researchers to contextualize their results by easily accessing literature that describes correlative microbial findings.

**Figure 3:**
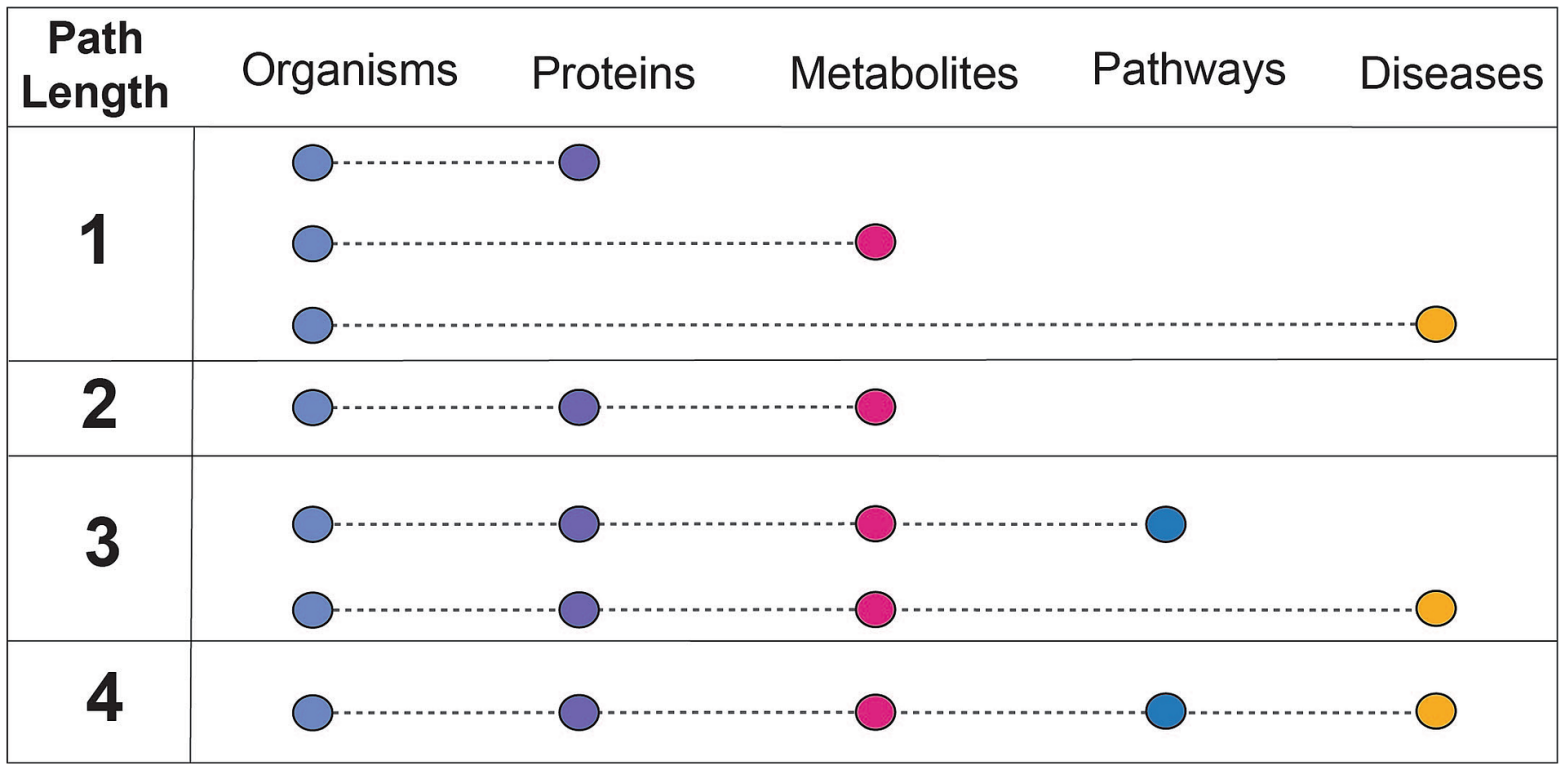
Understanding the connectedness of integrated databases based on path length. Path length refers to the number of relationships between unique concepts, or feature types, that are included within a resource. The feature types discussed in this context are microbes, proteins (or genes, human or microbial), metabolites (human or microbial), pathways (human or microbial), and diseases (human). The concept of path length is used to assess how comprehensively a resource can be used for mechanistic inference, or which relationships are needed from other databases to do so.

Findings of a specific experimental result can be related to a more complete mechanistic path by using relationships summarized in correlative databases. These databases have been used for corroboration of the findings of targeted experiments. For example, gutMGene has been used to corroborate the hypothesis that the gut microbial community plays an important role in cardiovascular disease through short chain fatty acid production by citing searchable microbe-metabolite relationships in the form of a network. Additionally, gutMDisorder has been used to validate polysaccharides identified to have a regulatory effect in disease through microbe-disease relationships in the form of a network (Hu et al., 2022; Wei et al., 2023). BugSigDB demonstrates the value in having a heterogeneous resource to explore patterns of microbial composition across studies, examines the commonly co-occurring or mutually exclusive individual or groups of microbes, and evaluates differences in microbial communities across body sites (Geistlinger et al., 2022). However, despite these highly useful applications of manually curated, correlative knowledge bases, there are key challenges that contribute to their limited use. A primary limitation of these databases is the small number of relationship types represented (designated as path length in Figure 3). Furthermore, it is difficult to align experimental results to such databases when concepts are not mapped to common primary knowledge sources, discussed more in challenges and future perspectives.

### 3.3 Mechanistic hypothesis generation

Hypothesis generation in microbiome research requires a diverse range of knowledge. To date, no resource or methodology supports the task of hypothesizing mechanisms of microbial processes that influence disease by including all categories of data described in Figure 3. However, some resources represent data in a way that supports inference, linking multiple complex relationships into a derived explanation. Structured, microbiome-relevant resources can support this automated inference. Knowledge graphs (KGs) are commonly used for this purpose due to their logical representation conducive to automated inference. KGs are simplified representations of related concepts through nodes (concepts) and edges (relationships between those concepts). The KG construction process involves the aggregation of content and harmonization to ontologies, most often through ingests that extract, transform, and load such information into a semantically consistent format. Graph-based models, the basis of KGs, enable complex queries and reasoning, which is especially useful for understanding the intricate interactions between microbes and the host. The following resources have varying levels of specificity to a particular disease, solely focus on microbial trait data, or lack the wider context necessary for disease-based inference.

MiKG4MD is one resource that represents how microbes are involved with mental disorders in the form of a knowledge graph (Liu et al., 2021). MiKG4MD was used to form specific queries that identify several sources describing the relationship between *Bifidobacterium dentium* and anxiety or depression via the neurotransmitter gamma-aminobutyric acid (GABA) (Liu et al., 2021). MiKG4MD has not been applied beyond the case studies that demonstrate its purpose, though these queries exemplify the hypothesis generating potential. The Pre-/Probiotics Knowledge Graph (PPKG) represents over 29,000 articles describing prebiotics and probiotics, combined with three other primary public databases, MeSH, UMLS and SNOMED CT (Table 1) (Liu et al., 2022). Similar to MiKG4MD, a specific query of PPKG showed 114 direct relationships identified between *Bifidobacterium bifidum* and disease, suggesting an influence on blood lipids, gut microbiome profiles, brain connectivity, and gene expression (Liu et al., 2022). KG-microbe is a resource that more broadly represents how microbes interact with their environment (Joachimiak et al., n.d.). KG-Microbe is useful to understand microbial traits and environments, such as soil or water as well as human anatomical sites, though it does not yet include information which connects microbes to disease. Several relevant ontologies that play an important role in the representation of the complex knowledge associated with the microbiome also exist. The ontology of host–microbe interactions (OHMI) is the only known OBO ontology resource that introduces a structured representation of microbe-host interactions (He et al., 2019). This resource makes a critical step in developing standards for how to represent host–microbe interactions through flexible and interoperable representations. Furthermore, OHMI aligns to several OBO ontologies including NCBI Taxonomy, the Environmental Ontology (ENVO), and the Uber-anatomy Ontology (UBERON). Importantly, OHMI does not include the mechanistic detail of proteins and metabolites necessary for inference, however the logical representation introduced can provide a framework for mechanistic inference (He et al., 2019). OHMI has not been updated since the original publication. OHMI introduces over 1,000 terms including microbes, host–microbe interactions, and study details (He et al., 2019).

There are also frameworks that synthesize multi-omic content in a graph database or network representation. BioChem4j is one such framework that automatically ingests content from multiple ontologies and represents microbes and their functional traits using the UniProt API (Swainston et al., 2017). BioChem4j is therefore an extensible resource from which researchers can gather the enzymes and metabolites involved in microbial biochemical reactions that may occur in any environment. BioChem4j has been applied toward a pipeline for the discovery and optimization of biosynthetic pathways, specifically for understanding a range of industrial microorganisms. The pipeline examined flavonoid production pathways and an alkaloid pathway in *Escherichia coli* for the purposes of microbial engineering for chemical production (Carbonell et al., 2018). The Unified Functional Network (UniFuncNet) is another framework that integrates multiple resources necessary for the construction of GSMNs (Queirós et al., 2022). The UniFuncNet framework can take as input a list of entities from different databases (e.g., proteins, genes, metabolites, etc) and output a network representation of all associations among such entities. UniFuncNet’s applications are demonstrated through two workflows which, for example, expanded existing GSMNs of *Akkermansia muciniphila* to include the biosynthesis and metabolism of glycans, or to relate compounds identified in a metabolomics dataset to relevant pathways and organisms (Queirós et al., 2022).

## 4 Challenges associated with the construction and applications of knowledge bases

### 4.1 Inconsistent taxonomy and metabolite nomenclature assignment

A major challenge arising from the availability of multiple taxonomic databases as well as multiple versions of the same taxonomic database are the resultant inconsistencies in the labeling of a microbe. The classification method of microbes curated from the literature is often overlooked, and in many cases a microbe may be assigned the wrong identifier (e.g., a microbe originally labeled via SILVA is assigned an NCBI Taxonomy identifier). Methods of taxonomic assignment in sequence-based studies of microbial population differ depending on whether small subunit (SSU) ribosomal RNA (rRNA) is targeted, also known as 16S sequencing for bacteria and archaea, or shotgun metagenomic sequencing is performed. Inconsistent classification, whether varying labels is due to lack of information or poor quality of sequencing reads, can impede the ability to relate findings about a given microbe across studies to each other and to their functional attributes, which is important for ultimately trying to understand microbe-host interactions at the mechanistic level. Additional challenges arise when microbial nomenclature is revised based on a better resolution of evolutionary relationships from sequencing data or phenotypic information, resulting in the same taxa having different names depending on the date of publication.

SILVA and Greengenes, which are built using sequences from the European Nucleotide Archive (ENA) and GenBank, respectively, are the most used taxonomic databases for 16S sequencing (Pruesse et al., 2007; Ceccarani and Severgnini, 2023; McDonald et al., 2023). SILVA uses a Bergey’s seed alignment (Garrity et al., 2004), then partially manually builds upon that classification to construct a phylogenetic tree which is used as a guide. In order to classify sequences, SILVA uses the SILVA Incremental Aligner (SINA) reference-based alignment tool for multiple sequence alignment, and assigns organism names according to the *Deutsche Sammlung fur Mikroorganismen und Zellkultren* (DSMZ) (Pruesse et al., 2012). In contrast to SILVA, which uses a pre-constructed tree, Greengenes constructs a *de novo* tree for taxonomic classification (DeSantis et al., 2006). Greengenes2 made significant updates by linking a substantial number of whole genome sequences from the International Nucleotide Sequence Database Collaboration (INSDC) (Arita et al., 2021), amplicons from the Living Tree Project (Yilmaz et al., 2014) and other resources, to create the largest tree with the broadest phylogenetic coverage to date (McDonald et al., 2023). A new version of the SILVA database is released semi-annually, whereas Greengenes2 only recently was released, 9 years after the prior version (Pruesse et al., 2007; McDonald et al., 2023). Although it is well established that use of different taxonomic databases and their versions can greatly impact taxonomic assignments made, there are limited solutions for dealing with this ambiguity when creating integrated resources.

Similar problems arise for the nomenclature of metabolites that are represented in manually curated databases. Main technologies used for metabolomics include mass spectrometry (MS)-based or nuclear magnetic resonance (NMR)-based approaches. Metabolomics can be approached with untargeted techniques (for hypothesis generation) or targeted techniques (for hypothesis testing) (Johnson et al., 2016). The naming and mapping of these metabolites therefore can introduce some uncertainty and similar discontinuity as microbial taxonomy. Metabolite identification is done by comparing the spectra obtained experimentally with that included in the curated knowledge bases or primary knowledge sources described above, such as ChEBI or ChEMBL (Hastings et al., 2016; Zdrazil et al., 2023). The mismatch of metabolite names and identifiers across these standardized resources, presents challenges for researchers to contextualize their findings and formulate hypotheses regarding their data using integrated resources (Merlet et al., 2016; Shaffer et al., 2017). Resources also exist that facilitate the classification relating their spectra to those of known metabolites to improve direct mapping such as the Global Natural Products Social molecular networking (GNPS) (Overbeek et al., 2014). The challenge of mismatching metabolite labels is especially prominent in the construction and alignment of GSMNs, which draw from these standardized databases. HMDB is one of the most comprehensive resources of known host and microbiome associated metabolites, still only representing a fraction of the metabolome, that cross-links many of standard chemical databases and identifiers to make this process more straightforward (Wishart et al., 2022). MetaNetX further facilitates mapping experimental results to representations in GSMNs to contextualize metabolomics findings (Moretti et al., 2021). An important direction of understanding microbiome-host relationships is evaluating how the microbiome and the metabolome interact with exogenous factors, such as diet, collectively called the exposome (Shaffer et al., 2017). The VMH is an important resource for this, as it introduces known relationships between the exposome and the metabolome (Noronha et al., 2019). Nomenclature challenges also are confronted in constructing correlative knowledge bases, such as gutMGene, in that chemical names that are manually curated, or text mined potentially, cannot be mapped to an identifier in a primary knowledge source (Cheng et al., 2022). As such, what exists in these resources may not accurately represent what was found in the corresponding study. The increased utility and standardization of naming of integrated knowledge bases is critical for addressing the challenges described, as integrated resources provide expansive knowledge that will support mechanistic exploration.

### 4.2 Semantic standardization

Another limitation of these resources is the extent to which entities are mapped to existing primary knowledge sources (e.g., ChEBI). Without mappings to a semantic standard, it is impossible to combine a resource with others as the concepts represented are not identical. Mechanistic curated knowledge bases such as KEGG, which introduce new identifiers due to their broadly represented and cross-linked information, are particularly useful resources to map to because of their scale and connectivity to other resources. Integrated knowledge bases play an important role in enabling one to search a broader field of knowledge, or relationships between more concept types (a higher path length as defined in Figure 3). The benefits of standardizing to ontologies are two-fold; first, ontologies offer a full hierarchy of relationships in a machine-readable format. Ontologies are curated by experts in both data engineering and the scientific area represented and provide a logical interpretation of knowledge categories. This makes it possible to abstract or concretize a concept depending on the mechanistic detail desired. Experimental results can be mapped to ontological concepts as an exact term (e.g., AKT-interacting protein isoform 2) or more broadly characterize the concept to a parent term (e.g., AKT-interacting protein). Second, KGs built of the logical representation of concepts in ontologies can be used to contextualize scientific results and infer mechanistic explanations. More comprehensive KGs can be constructed when all the knowledge represented in these resources is correctly mapped to useful ontologies.

Microbiome relevant knowledge bases primarily lack standardization in microbial and disease categories. The few databases that incorporate human diseases are limited in their degree of standardization. Ontologies such as the Monarch Disease Ontology (MONDO) and the Human Phenotype Ontology (HPO) have been developed as part of the Monarch initiative and provide logically coherent hierarchical representations of concepts (Köhler et al., 2021; Vasilevsky et al., 2022). MONDO, which includes resources such as Online Mendelian Inheritance in Man (OMIM) and Orphanet, is updated monthly, and introduces thousands of diseases and disorders. Mappings to resources such as MONDO support the applications of aggregate databases toward understanding microbial mechanisms in human disease. Microbes in gutMGene, gutMDisorder, Disbiome, Amadis, and GIMICA are mapped to NCBI Taxonomy, however those in MDAD and NJS16 are not (Figure 2). MDAD includes protein mappings to UniProt and metabolite mappings to DrugBank, while NJS16 only includes metabolite mappings to KEGG (Goodfellow et al., 2009; wwPDB consortium et al., 2019). The absence of mappings to NCBI Taxonomy or any structured phylogenetic database limits usability due to the inconsistencies in naming and taxonomic classification strategies. It is important that new resources map to the primary sources most often referenced by current integrated resources, as shown in Figure 2A by the colored node size, to ensure that concepts can be consistently identified. These standardization challenges limit the capacity to integrate sources of knowledge and make mechanistic claims using such knowledge.

### 4.3 Access methods and source characteristics of resources

The source characteristics of integrated resources can influence both their comprehensiveness and accuracy. Manually curated resources can have increased accuracy as content is provided through curation by experts directly from literature. While manual curation is nearly always at play due to the requirement of specific expertise in understanding microbes, the field is clearly approaching a new era of automated content extraction. Text mining approaches make this task more efficient, allowing for more content to be easily accessed by scientists with a wide variety of research interests.

Knowledge bases can be accessed in many ways depending on the type of users that they serve (Figure 1B). Wet-lab focused researchers interested in accessing the broad store of knowledge offered by these resources are primarily interested in interactive web interfaces. Curated knowledge bases KEGG and MetaCyc each offer interactive visual interfaces and useful pathway diagrams. Integrated knowledge bases such as MiMeDB and MACADAM also offer an interface to easily query the desired content, though not the same support in pathway diagrams as Wikipathways and Reactome. Reactome is even more uniquely suited to show interactive cartoon diagrams which can greatly increase accessibility to all users. Other programmatic ways of accessing these resources are important for the analyses that bioinformaticians do using complex datasets. The Simple Protocol And Resource Description Framework Query Language (SPARQL) is a query language for the Resource Description Framework (RDF), a framework that supports relationship-based data made available on the web (Candan et al., 2001). When SPARQL queries are supported, whether programmatically or via an API, computational users can easily access information through highly specialized queries. API support also enables this functionality.

Many mechanistic curated and integrated knowledge bases are offered as relational databases or tables, which supports fast access to a range of knowledge. Graph databases require traversal across a wider domain of information, and therefore are not quick in retrieval. However, graph databases can host information to a greater level of detail. For example, the “glycolysis” pathway in KEGG and MetaCyc host fewer than 30 metabolites or gene/gene products, whereas Reactome includes over 40 metabolites and 100 proteins. In relational databases such as KEGG, the detail comes in the nodes (genes, metabolites, organisms) and the relationships represent some interaction or input/output more generally. In graph databases such as Reactome, the edges provide a hierarchical set of content in themselves with much more detail, for example the edge “ADPGK:Mg2+ phosphorylates Glc to G6P” connects alpha-D-Glucose to alpha-D-glucose 6-phosphate. All reactions are rooted in literature evidence, providing a detailed account of biological interactions.

The formal representation of knowledge introduced by KGs can include heterogeneous biological content that is flexible and interoperable. The network structure of a KG supports inference based on both the semantic representation of knowledge and the structure of the graph, allowing one to infer new edges (hypothesized relationships between distinct concepts) or classify biological concepts. An important consideration for KGs is the model used to represent such complex knowledge. A logical semantic representation is critical for inference, and this can be difficult with such complex concepts as microbe-host interactions. It is generally useful to follow a predefined schema for interoperability and introducing new information, such as the Web Ontology Language (OWL) or the Biolink model (Bechhofer, 2009; Unni et al., 2022). These models allow harmonization of data sources across all knowledge types, which is especially important in the multi-omic nature of microbiome science. The types of edges within MiKG4MD are arbitrary and do not align with previously existing repositories, such as the Biolink Model or the Relationships Ontology (RO), both of which provide some standardized structure to the organization of a KG (Smith et al., 2005; Liu et al., 2021; Unni et al., 2022). However KG-microbe does align to the Biolink schema, which ingests microbial trait databases and combines them with ontologies such as ChEBI and GO (Joachimiak et al., n.d.). This was done using automated graph construction libraries that are a part of KG-Hub. Through some manual curation, KG-Microbe includes specific microbial traits to be represented in a way that aligns with the Biolink schema (Joachimiak et al., n.d.). It is important for the chosen schema to support interoperability between KGs, incorporation of any ontology or primary knowledge source, and correctly represent the heterogeneous data types necessary within a microbiome-relevant KG.

## 5 Future perspective

By indexing and linking multi-omic knowledge, integrated resources can contextualize results at the systems-level, corroborating findings from experimental observations, and provide promise toward uncovering novel hypotheses. We evaluate key categories of microbiome-relevant knowledge including microbes, host and microbial proteins, host and microbial metabolites, host and microbial pathways, and host diseases and argue that the extent to which these categories are covered by such integrated resources influences their ability to be adopted for mechanistic inquiry. It is important for users to evaluate the resource based on the six categories present here and their affordances (Figure 1A); ontologies and taxonomies, annotated databases, mechanistic curated knowledge bases, integrated knowledge bases, correlative curated knowledge bases, and inference-ready knowledge bases, in order to derive the best applications of such resources. We have also evaluated the primary traits of these resources including access methods, content, and source characteristics (summarized in Supplementary Table 1). The access points of the knowledge contained in these resources, whether programmatically or via a user-friendly web interface, can greatly affect the adoption by the intended user. Ensuring they support downloadable flat files or APIs translates to more readily available content for automatic hypothesis generation. Mapping the concepts represented in each resource is an important factor to consider in utilizing these resources, as it can limit the capacity for connecting it with other resources. Many correlative knowledge bases, for example, lack the level of nomenclature standardization to commonly used primary knowledge sources that is essential for wide adoption and integration of such resources. Future resources should always keep the nomenclature limitations in mind during construction and ensure that the level of standardization supports the intended use case.

Inference-ready knowledge bases such as KGs serve an important purpose in the microbiome field in supporting mechanistic hypothesis generation using existing knowledge. As shown, there are few resources that adequately map all categories of knowledge mentioned to enable explanations for microbe-disease associations to be understood. A focus on this connectedness, highly dependent on the level of standardization discussed previously, will drive the microbiome field toward a deeper understanding of microbe-host interactions via automated inference (Figure 2). Furthermore, it is important that these KGs use a data model that is highly interoperable and flexible to integrate heterogeneous data types. Applying these resources to mechanistic inference can help assess health outcomes and derive new understandings of multi-omic data sets through many methodologies such as linear modeling or machine learning based approaches. While these methodologies are not addressed in great detail, it is important to recognize their complexities.

Through this review of resources, we have provided evidence of the efforts to consolidate the rapidly increasing number of experimental findings surrounding the microbiome. We have published data resource mappings in a git-hub repository to ensure reproducibility and to support updates.^1^ We recognize that this review does not capture all possible resources, therefore encourage contributions to this repository in hopes of maintaining a useful source of information for researchers to select the most appropriate knowledge sources. We argue that the adoption of these resources and contributions to the field will be maximized with further standardization and connectedness. The application of these resources to understanding microbe-host-disease related questions holds promise for advancing biomedical understanding.

## Data availability statement

The repository used for developing the figures can be found in our git-hub repository https://github.com/lozuponelab/knowledge-source-mappings.

## Author contributions

BS: Conceptualization, Investigation, Visualization, Writing – original draft, Writing – review & editing. MA: Conceptualization, Investigation, Writing – review & editing. AC: Conceptualization, Investigation, Writing – review & editing. CM: Conceptualization, Investigation, Software, Visualization, Writing – review & editing. JS: Conceptualization, Investigation, Writing – review & editing. EW: Conceptualization, Investigation, Writing – review & editing. MPJ: Conceptualization, Investigation, Writing – review & editing. LH: Conceptualization, Investigation, Writing – review & editing. CL: Conceptualization, Investigation, Writing – review & editing.

## Footnotes

^1^https://github.com/lozuponelab/knowledge-source-mappings
