# Supplementary material for: Integrating biological knowledge for mechanistic inference in the host-associated microbiome
Source: Front Microbiol. 2024 Apr 4;15:1351678. doi: 10.3389/fmicb.2024.1351678 (PMC11024261; doi:10.3389/fmicb.2024.1351678)
Supplement: Supplementary file 1 [file Data_Sheet_1.PDF]

Supplementary

Supplementary Table 1. Attributes of all integrated resources discussed.

Resource	Structure	Accessibility	Path Length	Source	Curation Method	Update Frequency
**Mechanistic Curated Knowledge Bases**						
KEGG(20)	Relational database	• Web interface<br>• Downloadable files<br>• API	4	Literature	Manually curated	3mo
MetaCyc(19)	Relational database	• Web interface<br>• Downloadable files<br>• API	4	Literature	Manually curated	2-6mo
Reactome(47)	Graph database	• Web interface<br>• Downloadable files<br>• API	4	Literature	Manually curated - community driven	3mo
WikiPathways(48)	Graph database	• Web interface<br>• Downloadable files<br>• API	4	Literature	Manually curated - community driven	<3mo (content)<br>4y (structure)
HMDB(40)	Table	• Web interface<br>• Downloadable files	2	Literature	Manual curation	3-6y
BiGG(31)	Table	• Web interface<br>• Downloadable files<br>• API	2	Literature, multi-omic databases	Manually curated, automated extraction	4-6y
BacDive(45)	Table	• Web interface<br>• Downloadable files<br>• API	3	Literature, multi-omic databases	Manually curated, semi automated workflow	<3mo (based on connected resources)<br>2-3y (structure)
**Integrated Knowledge Bases**						
MiMeDB(38)	Table	• Web interface<br>• Downloadable files<br>• API	4	Multi-Omic databases	Manually curated, automated extraction	NA (only 1 known)
VMH(34)	Table	• Web interface<br>• Downloadable files<br>• API	4	Literature, multi-omic databases	Manually curated, automated extraction	NA (only 1 known)
MetaNetX(32)	Table	• Web interface<br>• Downloadable<br>• API	2	Literature, multi-omic databases	Manually curated, automated extraction	2-5y
MACADAM(39)	Relational SQLite db/table	• Web interface<br>• Downloadable files<br>• API	3	Literature, multi-omic databases	Automated extraction	6mo

BioModels(33)	table	• Web interface<br>• Downloadable files<br>• API	2	Literature	Manually curated, automated extraction	<3mo (content)<br>2-4y (structure)
KBase(35)	Table	• Web interface<br>• API	4	Multi-omic databases	Automated extraction	3mo (database and apps)
PATRIC(46)	Table	• Queries<br>• Web interface<br>• Downloadable files<br>• APIs	4	Multi-omic databases	Automated extraction	6mo
**Correlative Curated Knowledge Bases**						
MDAD(49)	Table	• Web interface<br>• Downloadable files	2	Literature	Manual curation	NA (only 1 known)
gutMGene(50)	Table	• Web interface<br>• Downloadable files	2	Literature	Manual curation	1y (once)
gutMDisorder(51)	Table	• Web interface<br>• Downloadable files	1	Literature	Manual curation	3y (once)
Amadis(53)	Table	• Web interface<br>• Downloadable files	1	Literature	Manual curation	NA (only 1 known)
Disbiome(52)	Table	• Web interface<br>• Downloadable files	1	Literature	Manual curation	3mo
GIMICA(54)	Table	• Web interface<br>• Downloadable files	1	Literature	Manual curation	NA (only 1 known)
BugSigDB(59)	Table	• Web interface<br>• Downloadable files	1	Literature	Manual curation, community-driven	<3mo (content)
dbBact(55)	Table	• Web interface<br>• Downloadable files	1	Literature	Manual curation	NA (only 1 known)
NJS16(56)	table	• Downloadable files	1	Literature	Manually curated	NA (only 1 known)
**Inference-Ready Knowledge Bases**						
MiKG4MD(60)	Knowledge Graph	• Downloadable files	3	Literature	Manual curation	NA (only 1 known)
Pre-Probiotics KG(61)	Knowledge Graph	• Downloadable files<br>• APIs	1	Literature, public databases	Manual curation, natural language processing	NA (only 1 known)

KG-Microbe(62)	Knowledge Graph	• Source code<br>• Downloadable files	2	Literature, public databases	Manual curation, natural language processing	NA (only 1 known)
Biochem4j(64)	Graph database	• Source code<br>• Downloadable files	4	Public databases	Automated	NA (only 1 known)

Supplementary Table 2. Acronyms used in the manuscript.

Concept	Definition
API	Application programming interface
BacDive	Bacterial Diversity Metadatabase
BiGG	Biochemical, Genetic and Genomic knowledge base
BLAST	Basic Local Alignment Search Tool
BRENDA	BRaunschweig ENzyme DAtabase
BV-VRC	Bacterial and Viral Bioinformatics Resource Center
CAZy	Carbohydrate Active Enzymes
ChEBI	Chemical Entities of Biological Interest
COGs	Cluster of Orthologous Groups
DAG	Directed Acyclic Graph
DB	Database
DSMZ	Deutsche Sammlung fur Mikroorganismen und Zellkultren
EC	Enzyme Commision
ENA	European Nucleotide Archive
ENVO	Environmental Ontology
FAPROTAX	Functional Annotation of Prokaryotic Taxa
GABA	gamma-aminobutyric acid
GIMICA	Host Genetic and Immune Factors Shaping Human Microbiota
GNPS	Global Natural Products Social molecular networking
GO	Gene Ontology
GSC	Genomic Standards Consortium
GSMN, GEM	Genome-Scale Metabolic Network
GTDB	Genome Taxonomy Database
HIV	human immunodeficiency virus
HMDB	Human Metabolome Database
HPO	Human Phenotype Ontology
ICD	International Classification of Diseases
IJSEM	International Journal of Systematic and Evolutionary Microbiology
INSDC	International Nucleotide Sequence Database Collaboration

KBase	Department of Energy Systems Biology Knowledgebase
KEGG	Kyoto Encyclopedia of Genes and Genomes
KG	Knowledge graph
KOALA	KEGG Orthology And Links Annotation
MACADAM	MetAboliC pAthways DAtabase for Microbial taxonomic groups
MDAD	Microbe-Drug Association Database
MeSH	Medical Subject Headings
MIMARKS	Minimum Information about Marker Gene Sequence
MiMeDB	Human Microbial Metabolome Database
MiRBase	microRNA sequence database
MIxS	Minimum Information about any (x) Sequence
MONDO	Monarch Disease Ontology
MS	Mass Spectrometry
NCBITaxon	NCBI Taxonomy
NMR	Nuclear Magnetic Resonance
OBO	Open Biological and Biomedical Ontologies
OHMI	Ontology of Host-Microbe Interactions
OMIM	Online Mendelian Inheritance in Man
OWL	Web ontology language
PATRIC	Pathosystems Resource Integration Center
PD	Parkinson’s disease
PDB	Protein Data Bank
PEDANT	Protein Extraction, Description and Analysis Tool
PGDB	pathway genome database
PIR/PSD	Protein Information Resource/Protein Sequence Database
PMID	PubMed Identifier
PPKG	Pre-/Probiotics Knowledge Graph
PRO	Protein Ontology
PW	Pathway
RDF	Resource Description Framework

RO	Relations ontology
SMPDB	Small Molecule Pathway Database
SNOMED CT	Systematized Nomenclature of Medicine-Clinical Terminology
SPARQL	Simple Protocol And Resource Description Framework Query Language
SSU rRNA	Small Subunit Ribosomal RNA
UBERON	Uber-anatomy ontology
UMLS	Unified Medical Language System
UniFuncNet	Unified Functional Network
VMH	Virtual Metabolic Human
